# Physiological Loading Can Restore the Proteoglycan Content in a Model of Early IVD Degeneration

**DOI:** 10.1371/journal.pone.0101233

**Published:** 2014-07-03

**Authors:** Rahul Gawri, Janet Moir, Jean Ouellet, Lorne Beckman, Thomas Steffen, Peter Roughley, Lisbet Haglund

**Affiliations:** 1 Orthopaedic Research Laboratory, McGill University, Montreal, Quebec, Canada; 2 Shriners Hospital for Children, McGill University, Montreal, Quebec, Canada; 3 McGill Scoliosis and Spine Group, Montreal, Quebec, Canada; Ohio State University, United States of America

## Abstract

A hallmark of early IVD degeneration is a decrease in proteoglycan content. Progression will eventually lead to matrix degradation, a decrease in weight bearing capacity and loss of disc height. In the final stages of IVD degradation, fissures appear in the annular ring allowing extrusion of the NP. It is crucial to understand the interplay between mechanobiology, disc composition and metabolism to be able to provide exercise recommendations to patients with early signs of disc degeneration. This study evaluates the effect of physiological loading compared to no loading on matrix homeostasis in bovine discs with induced degeneration. Bovine discs with trypsin-induced degeneration were cultured for 14 days in a bioreactor under dynamic loading with maintained metabolic activity. Chondroadherin abundance and structure was used to confirm that a functional matrix was preserved in the chosen loading environment. No change was observed in chondroadherin integrity and a non-significant increase in abundance was detected in trypsin-treated loaded discs compared to unloaded discs. The proteoglycan concentration in loaded trypsin-treated discs was significantly higher than in unloaded disc and the newly synthesised proteoglycans were of the same size range as those found in control samples. The proteoglycan showed an even distribution throughout the NP region, similar to that of control discs. Significantly more newly synthesised type II collagen was detected in trypsin-treated loaded discs compared to unloaded discs, demonstrating that physiological load not only stimulates aggrecan production, but also that of type II collagen. Taken together, this study shows that dynamic physiological load has the ability to repair the extracellular matrix depletion typical of early disc degeneration.

## Introduction

The intervertebral discs (IVD) of the spine function as shock absorbers and allow for flexibility and movement of the head and upper body [1]. The IVD is a complex and non-homogenous tissue with two distinct regions, the central nucleus pulposus (NP) and the surrounding annulus fibrosus (AF). The IVD is attached to the vertebral bodies of the spine via cartilaginous endplates at the top and bottom so creating a continuous unit.

The major components of the IVD extracellular matrix are proteoglycans and collagens.

A young and healthy NP is gelatinous with a very high proteoglycan content. The collagen in the NP is mainly type II fibres forming a randomly organised network. The AF is more fibrous with lower proteoglycan content. The collagen, mainly type I, is arranged into highly organized structures forming strong concentric rings. Aggrecan is the major proteoglycan in the IVD and the many glycosaminoglycan chains attached to its core-protein are highly anionic and attract and retain water. Aggrecan is entrapped in the collagen network that limits disc swelling and a combination of the two molecules provides for resistance to compression. A number of non-collagenous molecules are also found in the extracellular matrix of the IVD. Among these are the leucin-rich-repeat (LRR) family of proteins, including chondroadherin (CHAD) [2]–[4]. CHAD is found throughout the extracellular matrix of the IVD. It interacts with collagen and is known to link the cells to the extracellular matrix and may play a role in promoting matrix homeostasis [5]–[8]. The IVD extracellular matrix is altered as a result of ageing and degeneration. Aggrecan is fragmented and the amount is reduced, as a result of normal aging [4], [9]. Whereas, CHAD is intact in the healthy IVD at all ages but is fragmented as a result of degeneration [10], [11]. The rate of aggrecan fragmentation is increased in degeneration and its loss is one of the hallmarks of IVD degeneration. The loss of aggrecan from the NP lowers the resistance to compression which results in reduced disc height and altered mechanical properties of the IVD [1].

A sparse cell population is located in the vast disc extracellular matrix [12], with a distinct cell type populating each region of the IVD. The NP is populated by chondrocyte-like NP cells, the AF with more fibroblast-like AF cells and the cartilaginous endplates by chondrocytes. The number of cells decreases and cell senescence increases with age and degeneration, and this contributes to the difficulty in maintaining a functional matrix [13], [14]. Disc cells respond to mechanical loading depending on load magnitude, frequency and duration [15]–[17]. Loading influences both matrix turnover and cell viability [18], [19]. Both excessive dynamic and static compressive loading result in increased metalloproteinase synthesis and a decrease in cell viability and aggrecan content [20]–[26]. Physiological load is thought to be beneficial to disc health, but it is not clear to what extent it can influence matrix synthesis early in the degenerative process. It is crucial to understand the interplay between mechanobiology, disc composition and metabolism in order to provide exercise recommendations to patients with early disc degeneration and possibly also to patients treated for early disc degeneration with for example bioactive substances.

A degeneration protocol, along with a bioreactor that facilitates organ culture of intact discs in a controlled dynamically loaded environment, has been developed to address such questions [19], [27]. This study investigates the effect of physiological loading compared to no loading on matrix homeostasis in bovine discs with induced degeneration.

## Materials and Methods

### Source of reagents

The polyclonal rabbit antibody recognising type II collagen was from Abcam (Cambridge, MA, USA, ab300) and the polyclonal rabbit antibody recognizing chondroadherin was a kind gift from Dr. Dick Heinegård, Lund University, Sweden [28]. The secondary horseradish peroxidase (HRP)-conjugated anti-rabbit antibody was from Cell Signaling, New England Biolabs (Ipswich, MA, USA). The enhanced chemiluminescence (ECL) detection system was from Perkin-Elmer (Waltham, MA, USA). DTT was from Invitrogen/Life Technologies (Grand Island, NY, USA) and iodacetamide from Sigma-Aldrich (St Louis, MO, USA).

### Disc isolation

Bovine tails from 24- to 30-month-old steers were obtained from Boucherie B Poirier, Saint-Louis-de-Gonzague, Quebec Canada. The tails were dissected free of soft surrounding tissue, and pedicles for each segment were removed. The largest first 3–4 caudal discs were isolated with intact cartilage endplates as described by Jim *et al* [27]. Briefly, parallel transverse cuts were made through the vertebral bodies close to the cartilage endplates. The bone and adjacent calcified part of the cartilaginous endplate were then removed using a surgical round burr on a high-speed drill (ConMed, Racine, WI, USA). The discs were processed until the surface of the tissue was soft and flexible without detectable calcified tissue.

### Disc organ culture and degeneration

The discs were rinsed in PBS supplemented with 500 U/mL U/mL penicillin, 1000 µg/mL streptomycin (Gibco/Life Technologies, Grand Island, NY, USA) and 0.25 µg/mL fungizone (Gibco), then placed in culture chambers (sterile 80 mL specimen containers, STARPLEX Scientific, Etobicoke, Ontario) containing 50 mL culture medium (Dulbecco’s Modified Eagle Medium with 2 mM Glutamax and 25 mM Hepes, supplemented with 5% fetal bovine serum, 500 U/mL penicillin, 500 µg/mL streptomycin, 50 µg/mL l-ascorbate). Half of the media volume was changed twice a week. Forty-eight isolated discs were cultured without external load applied for 48 h (Table 1). Degeneration was induced by a single injection of trypsin (T8003, Sigma, 100 or 10 µg/50 µL PBS) into the center of 36 discs using a 28 G needle. Eighteen control discs were injected with the same volume of PBS. Thirty-six discs were cultured for an additional 48 h without external load and were then divided into three groups, bioreactor loaded, unloaded and stress profilometry (Table 1). Eighteen discs were loaded [19] and 18 were cultured without external load applied. The discs designated for cyclic loading were 48 h after trypsin or PBS injection placed under low static load (0.1 MPa) for an additional 48 h before cyclic dynamic loads ranging from 0.1–0.3 MPa was applied for 4 h/day. Six trypsin treated and 6 control discs were cultured for 7 days un-loaded. These discs were used for stress profilometry. The disc heights ranged from 8.8 to 11.3 mm (10.24±1 mm) and the diameter ranged from 23.4 to 30.7 mm (26.9±2.1 mm).

**Table 1:** Experimental setup.

Treatment	Bioreactor		Stress profilometry
	Loaded	Unloaded	Unloaded
100 µg trypsin	6	6	6
10 µg trypsin	6	6	0
PBS (control)	6	6	6

### Stress profilometry

The disc were placed between porous platen covering 50% of the disc cross-sectional area. Loading was carried out on a 858 Mini Bionix (MTS, Eden Prairie, MN, USA). The discs were preconditioned for 5 minutes at 0.1 MPa load, the load was then increased to 0.3 MPa and the pressure profiles were immediately then recorded. A needle (1.3 mm diameter) with a pressure sensor mounted 3 mm distant from the tip was inserted in the mid plane of the disc through the annulus, until the sensor was visibly outside of the opposite annular region [19], [29]. The needle was then coupled to a resistor-based linear position sensor and was then steadily and slowly withdrawn backwards across the disc along its insertion path. Positional data and pressure data was recorded and plotted. The average NP plateau pressures were calculated.

### Cell viability

Metabolic activity was measured in the discs after 14 days culture in loaded and un-loaded conditions. Two 1.5 mm sections were taken in the sagittal plane through the center of the discs using a custom made cutting tool [27]. Half of the NP region from one section was incubated in 1 mL culture medium containing 10% alamarBlue (Invitrogen) for 4 h. 3 times 100 uL of each sample was transferred to black 96 well plates (Corning), and fluorescence intensity was measured at excitation 540 nm, emission 585 nm (Tecan M200 Infinity Pro). 10% alamarBlue in culture medium was used as a blank value and was subtracted from all the sample values. The intensity was then normalized to the weight of the tissue.

### Extraction of ECM proteins and proteoglycans

The second half of the section was thinly sliced and proteins and proteoglycans were extracted on a wet weight per volume basis using 15 volumes extraction buffer (4 M guanidinium chloride, 50 mM sodium acetate, pH 5.8, 10 mM EDTA, COMPLETE (Roche Diagnostics, Laval, QC, Canada). The extracts was kept at 4°C under continuous agitation for 48 h and were then cleared by centrifugation at 16 000 g for 30 min.

### GAG analysis

Sulfated glycosaminoglycans (GAGs) were quantified from tissue extracts by the modified dimethyl methylene blue (DMMB) dye-binding assay described by Mort and Roughley [30]. Tissue extracts were diluted in water (1∶100). The standard (chondroitin 6 sulphate, Sigma) was prepared in water to have a final concentration ranging from 0.5 to 5 µg/mL. Sample or standard dilution (20 µL) was then mixed with 180 µL of DMMB solution and the absorbance was measured at 530 nm (Tecan M200 Infinity Pro). Samples and standards were adjusted to contain the same amount of extraction buffer.

### Agarose gel electrophoresis

Proteoglycans in 10 µL aliquots of disc extracts were precipitated by the addition of 9 volumes of 95% ethanol; the pellets were washed twice in 95% ethanol, and finally lyophilized and dissolved in distilled water. The samples were reduced, alkylated and mixed with sample buffer (0.1 M Tris-HCl, 0.768 M glycine, 0.01% Bromophenol blue, 50% sucrose, 0.05% SDS, pH 8.3) and boiled for 10 minutes. The proteoglycans were separated by electrophoresis in 1.2% agarose gels [31]. The gel was stained with 0.02% (w/v) Toluidine blue in 3% acetic acid with 0.5% (w/v) Triton X 100, and destained with 3% acetic acid then distilled water.

### Western blot analysis

Proteins and proteoglycans in 40 µL aliquots of disc extracts were precipitated in 9 volumes of 95% ethanol, the pellets were washed twice in 95% ethanol, and finally lyophilized. Samples were re-dissolved in 200 µL SDS sample buffer and 20 µL of this was separated by SDS-PAGE (4–12% NOVEX gels) under reducing conditions. Separated proteins were transferred to nitrocellulose membranes and western blotting was performed using antibodies recognizing type II collagen and chondroadherin. The membranes were blocked with 3% BSA in 10 mM Tris-HCl, pH 7.4, 0.15 M NaCl, and 0.2% Tween (blocking buffer) and then incubated with the primary antibodies at a 1∶1000 (CHAD) and 1∶5000 (type II collagen) dilution in blocking buffer containing 3% BSA followed by the secondary antibody conjugated with HRP (1∶2000 dilution) in blocking buffer containing 1% BSA. The bound antibody was visualized by chemiluminescence (GE Healthcare) using the ImageQuant LAS4000 (GE Healthcare, Baie d’Urfe, QC, Canada). ImageQuant TL array analysis software was used to analyze the blots. To generate data for the graphs, western blotting was performed twice for each sample run on separate membranes; two reference extracts were included on each blot. The relative quantity of each protein was extrapolated using the reference samples and negative controls included on each blot. For the blots displayed, the 6 samples per group were combined and analysed as a pool.

### Histology

The NP region of the second 1.5 mm section was fixed between porous platens in 4% paraformaldehyde, lysine, periodate as described by McLean and Nakane [34] for 24 h before transferring to decalcifying solution (10% EDTA in 0.1 MTris-HCL, pH 7.4) for 12 days with solution changes twice daily. Samples were then embedded in paraffin and 5 µm thick sections were prepared and stained with haematoxylin and Safranin O-fast green [32].

### Statistical analysis

T-test was used to compare the groups as indicated in the figures. One way ANOVA with Tukey’s multiple comparison test was used for comparing multiple groups.

## Results

**Figure 1:**
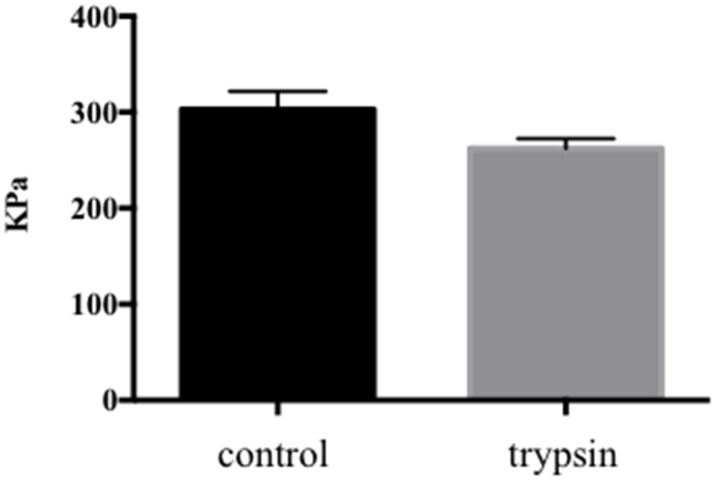
Stress profilometry. Comparison of average plateau pressures for control and trypsin-treated discs (p 0.07).

Proteoglycan loss is a hallmark of early disc degeneration and trypsin injection has previously been used to mimic this in bovine discs [27]. In this study, 10 or 100 ug trypsin per disc was injected into the center of the discs. The aim was to induce aggrecan depletion without disrupting the collagen network. A disruption of the collagen network could result in severely decreased weight bearing capacity and in uneven load-curves with high peak loads already at low load magnitudes. Stress profilometry was used to evaluate the load profile at 0.3 MPa load, which was the load magnitude planned for this study. This load magnitude has previously been shown to be optimal for maintained cell viability in non-degenerate bovine discs cultured under dynamic loading for up to 4 weeks [19]. The load curves followed the same general profile with an average plateau pressure of 303±19 kPa in the control discs and 262±10 kPa in the discs treated with 100 ug of trypsin (Figure 1) (p 0.07). The lower pressure measured in the trypsin-treated discs is indicative of mild degeneration.

**Figure 2:**
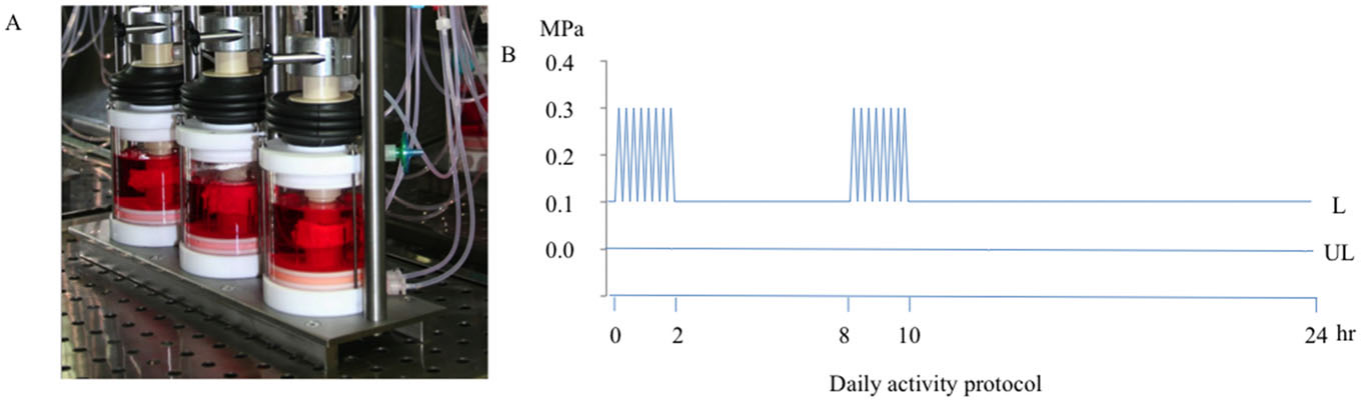
Culturing of intact loaded and unloaded IVDs. **A**, The bioreactor system consists of two subsystems: culture chamber and loading frame. The IVDs are placed between porous platens attached to a piston. Media is circulated in through the top and bottom platens. The loading frame is a triple unit, with an pneumatic actuator controlled with a proportional pressure control valve. A load cell and linear variable displacement transformer measure load and displacement. The actuator is connected to the piston of the culture chamber. The system is computer controlled. **B**, The discs were cultured for 14 days unloaded (UL) or loaded (L) at 0.1 Hz as illustrated.

**Figure 3:**
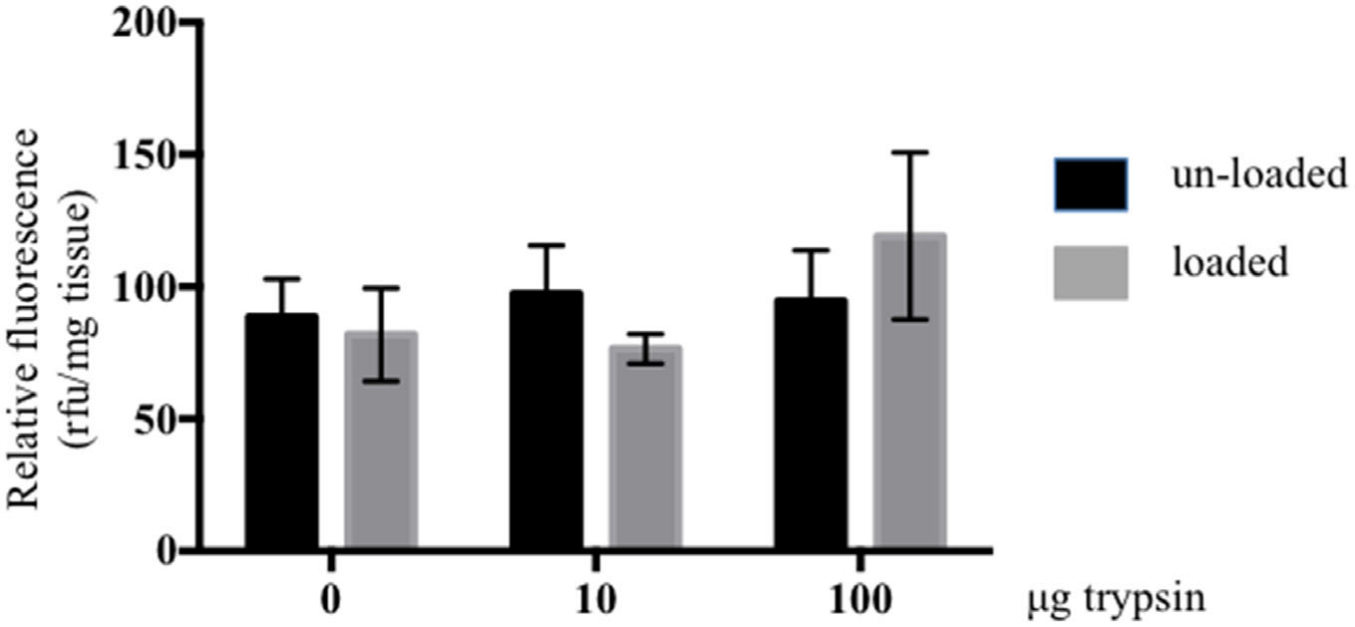
Metabolic activity in loaded and unloaded discs. Metabolic activity was evaluated by the alamarBlue assay in NP tissue of loaded and unloaded IVDs after 14 days of culture. The IVDs were injected with 0, 10 or 100 µg trypsin prior to loading. Relative fluorescence was normalized to tissue weight (n 6, error bars represent SEM).

Viable and metabolically active cells are necessary for matrix homeostasis or tissue remodelling. Although trypsin-treated discs only displayed a minor drop in pressure after one sequence of static load at 0.3 MPa, 14 days of dynamic loading of the same magnitude could eventually affect cell health adversely. Trypsin-treated and control discs were cultured for 14 days unloaded or under dynamic loading as indicated in Figure 2A–B [19]. Metabolic activity was evaluated with the Alamar blue assay. There was no statistically significant difference between unloaded and loaded control discs, or between unloaded and loaded trypsin-treated discs. There was also no statistically significant difference between the loaded trypsin-treated and control discs, indicating that viable metabolically active cells remained in the discs independent of treatment (Figure 3). We concluded from these results that the loading regime applied was within a physiological range.

**Figure 4:**
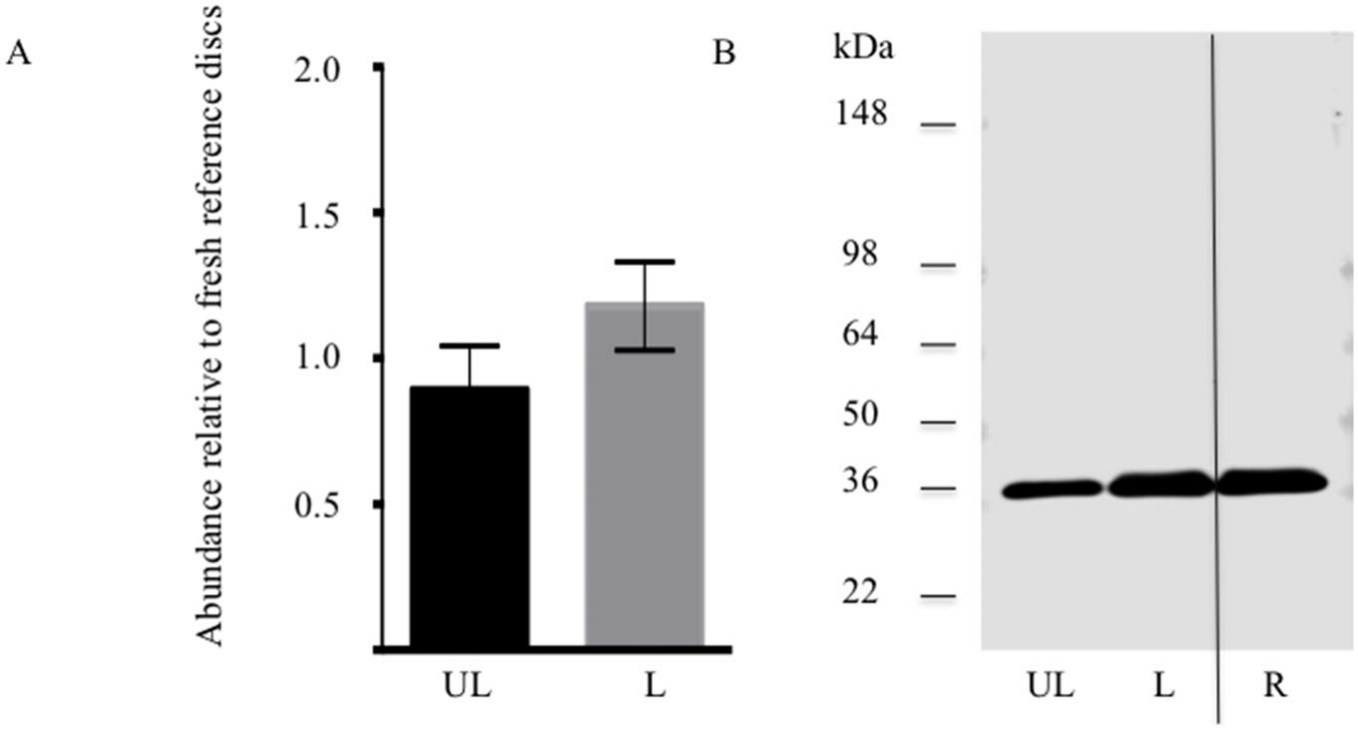
Extractable chondroadherin in loaded and unloaded discs. NP tissue of loaded (L) and unloaded (UL) IVDs were analysed by western blotting after 14 days of culture. The IVDs were injected 100 µg trypsin prior to loading. A, Semi-quantitative analysis of chondroadherin with a molecular weight of about 36 kDa. Each sample was evaluated in duplicate on 2 separate western blots. Band intensity was normalized to reference samples (R) included on each blot. B, representative Western blot of pooled extracts. (n 6 discs from different animals). Error bars represent SEM.

Maintained chondroadherin abundance and structure was used as to evaluate the integrity of the disc extracellular matrix. The quantity of chondroadherin extracted from the trypsin-treated loaded and unloaded discs was evaluated in comparison to reference samples of freshly isolated discs. A similar level of chondroadherin was found in the loaded samples compared to the reference (Figure 4). A non-significant increase was detected in trypsin-treated loaded discs compared to unloaded discs, further indicating that a functional matrix is maintained in the loaded discs.

**Figure 5:**
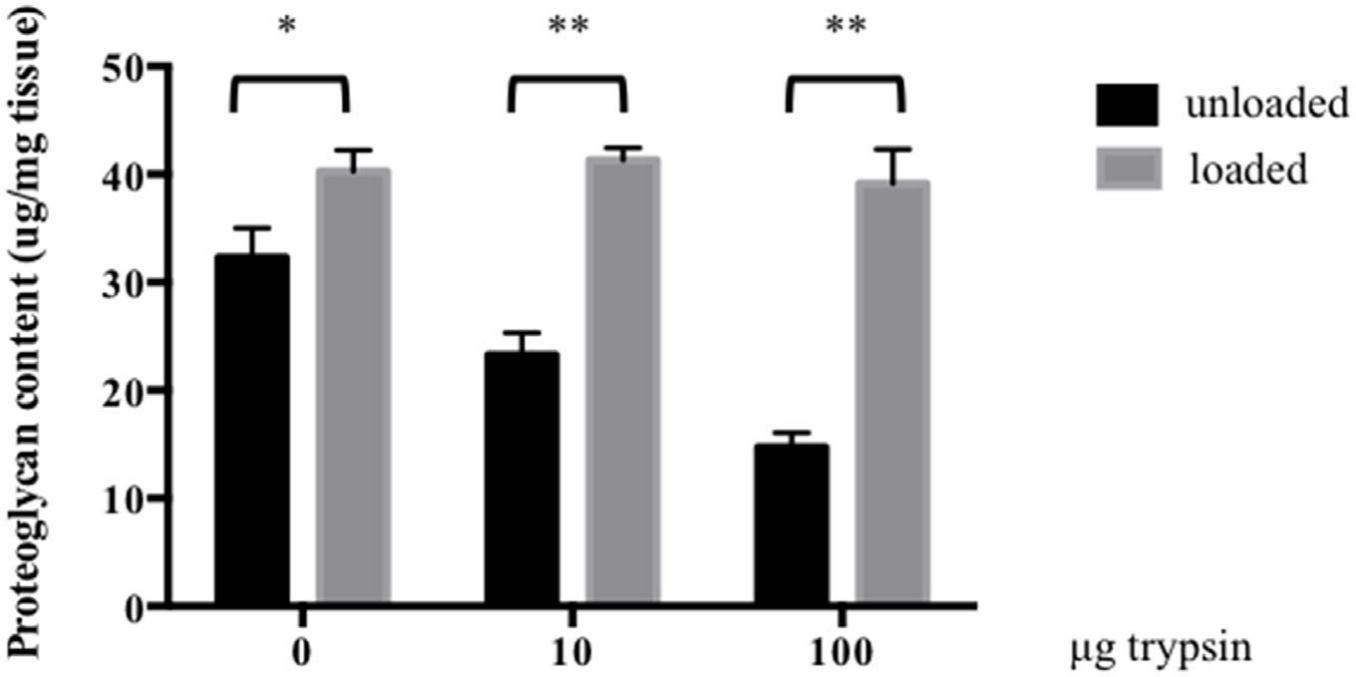
The influence of physiological load on proteoglycan synthesis in trypsin-treated discs. Proteoglycan content was evaluated by the DMMB assay in NP tissue of loaded and unloaded IVDs after 14 days of culture. The IVDs were injected with 0, 10 or 100 µg trypsin prior to loading. Proteoglycan content was normalized to tissue weight (n 6, error bars represent SEM) *p<0.05, **p<0.001.

As physiological load is thought to activate proteoglycan synthesis, the DMMB method was used to quantify proteoglycan content in the discs after 14 days of culture. The proteoglycan content was about 20% higher in control discs cultured under load compared to control discs cultured unloaded (p 0.04). In trypsin-treated discs cultured in the absence of load, proteoglycan content decreased by 30% and 60% when treated with 10 and 100 ug trypsin, respectively, compared to control discs. The proteoglycan content was decreased by 44% and 64% when unloaded trypsin-treated discs were compared to loaded discs with the same dose of trypsin treatment. The proteoglycan concentrations in loaded trypsin-treated discs were significantly higher than in unloaded discs (*p*<0.0001. No statistical difference in proteoglycan content was observed amongst loaded trypsin-treated and control discs (*p*>0.05). The results indicate that physiological load has the ability to restore proteoglycan content in discs with mild degeneration (Figure 5).

As no difference in proteoglycan restoration was found after treating with 10 and 100 ug trypsin, only the higher dose was evaluated for the remainder of the analysis.

**Figure 6:**
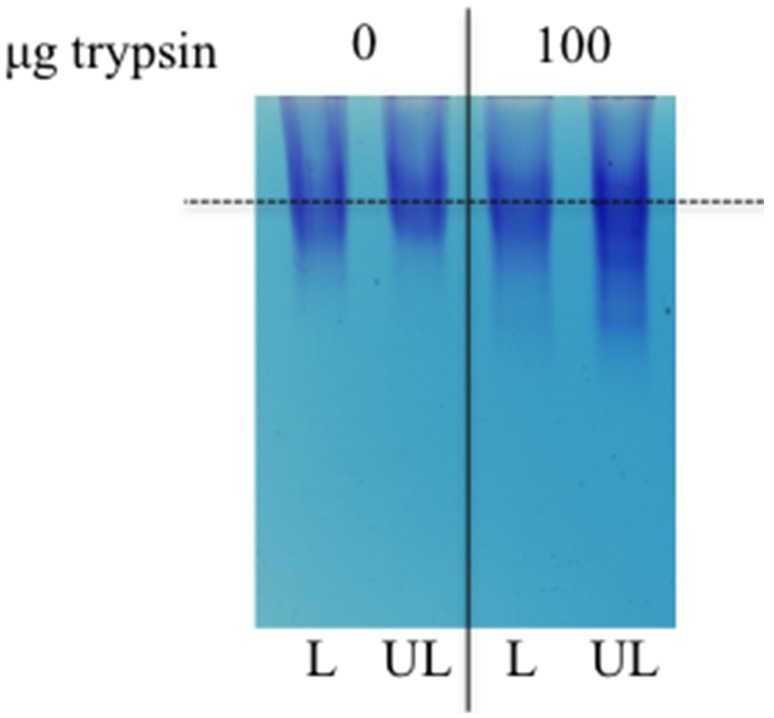
Proteoglycan composition in loaded and unloaded discs. Proteoglycan composition was evaluated by agarose gel electrophoresis in NP tissue of loaded (L) and unloaded (UL) IVDs after 14 days of culture. The IVDs were injected with 100 µg trypsin prior to loading. Equal amounts of GAG was loaded (n = 6 extracts per condition were pooled).

GAG content does not indicate if the proteoglycan structure is the same in the trypsin-treated loaded and unloaded discs. To address this, extracted proteoglycans were analyzed by agarose gel electrophoresis. Equal amounts of proteoglycans (as determined by DMMB) were loaded for each sample-pool. The size distribution was similar in the loaded controls and loaded trypsin-treated samples, whereas the proteoglycans in the unloaded trypsin-treated samples were more heterogeneous in size with more fragmented molecules (Figure 6). The data demonstrates that the newly synthesized proteoglycans in degenerate loaded discs are of the same size range as those of non-degenerate discs.

**Figure 7:**
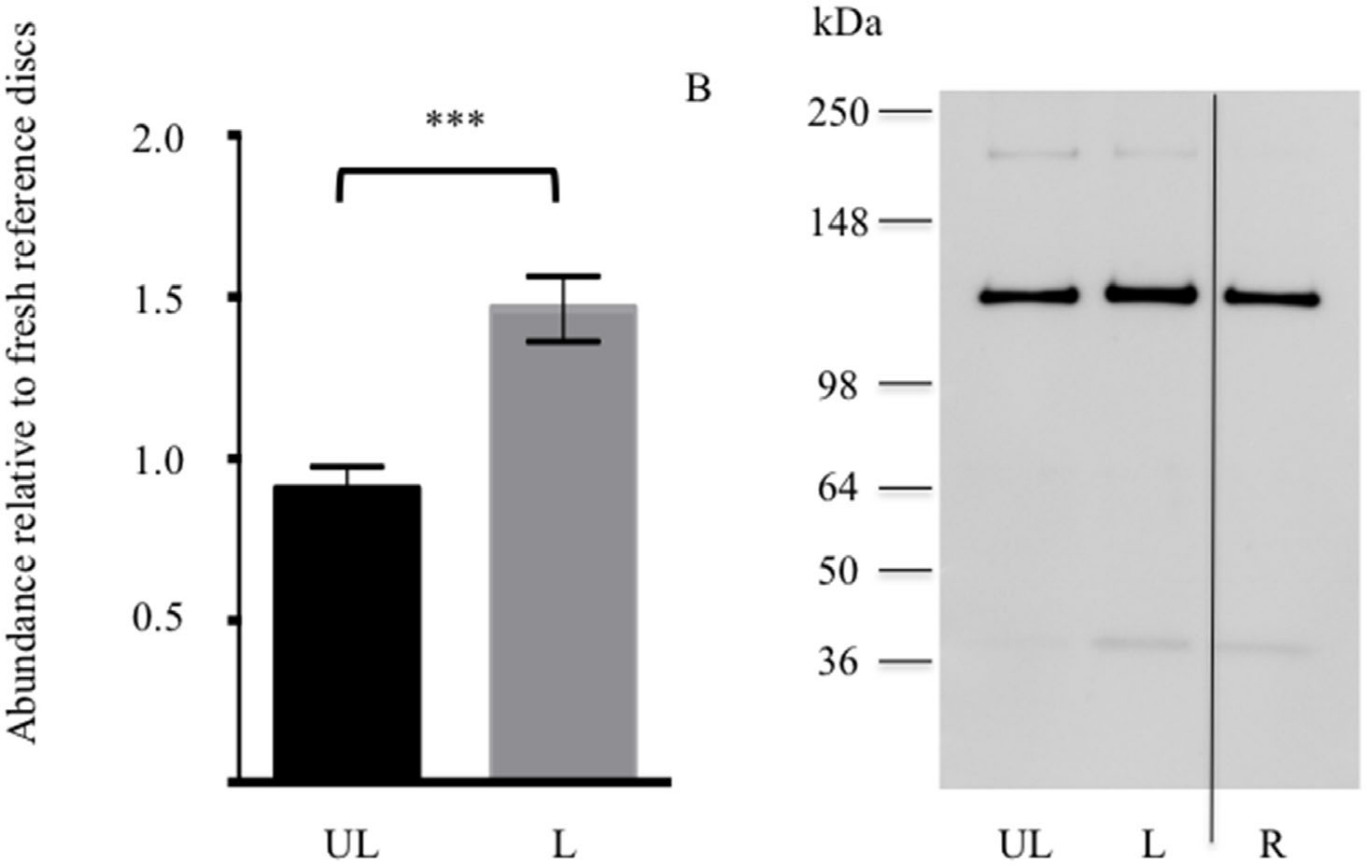
Newly synthesised extractable type II collagen in loaded and unloaded discs. NP tissue of loaded (L) and un-loaded (UL) IVDs were analysed by western blotting after 14 days of culture. The IVDs were injected 100 µg trypsin prior to loading. A, Semi-quantitative analysis of type II collagen with a molecular weight of about 135 kDa. Each sample was evaluated in duplicate on 2 separate western blots. Band intensity was normalized to reference samples (R) included on each blot. B, representative western blot of pooled extracts. (n 6 discs from different animals, p<0.0001). Error bars represent SEM.

Restoration of a functional extracellular matrix requires new collagen synthesis in addition to proteoglycan. Newly synthesised type II collagen is not cross-linked in the tissue and is therefore extractable. Therefore, the levels of extractable type II collagen were assessed (Figure 7). The quantity of type II collagen extracted from the trypsin-treated loaded and unloaded discs were evaluated in comparison to reference samples of freshly isolated discs. A similar level of type II collagen was found in the unloaded samples as compared to the reference. Significantly more extractable type II collagen was detected in trypsin-treated loaded discs compared to unloaded discs (*p*<0.001). Thus physiological load not only stimulates aggrecan production, but also that of type II collagen.

**Figure 8:**
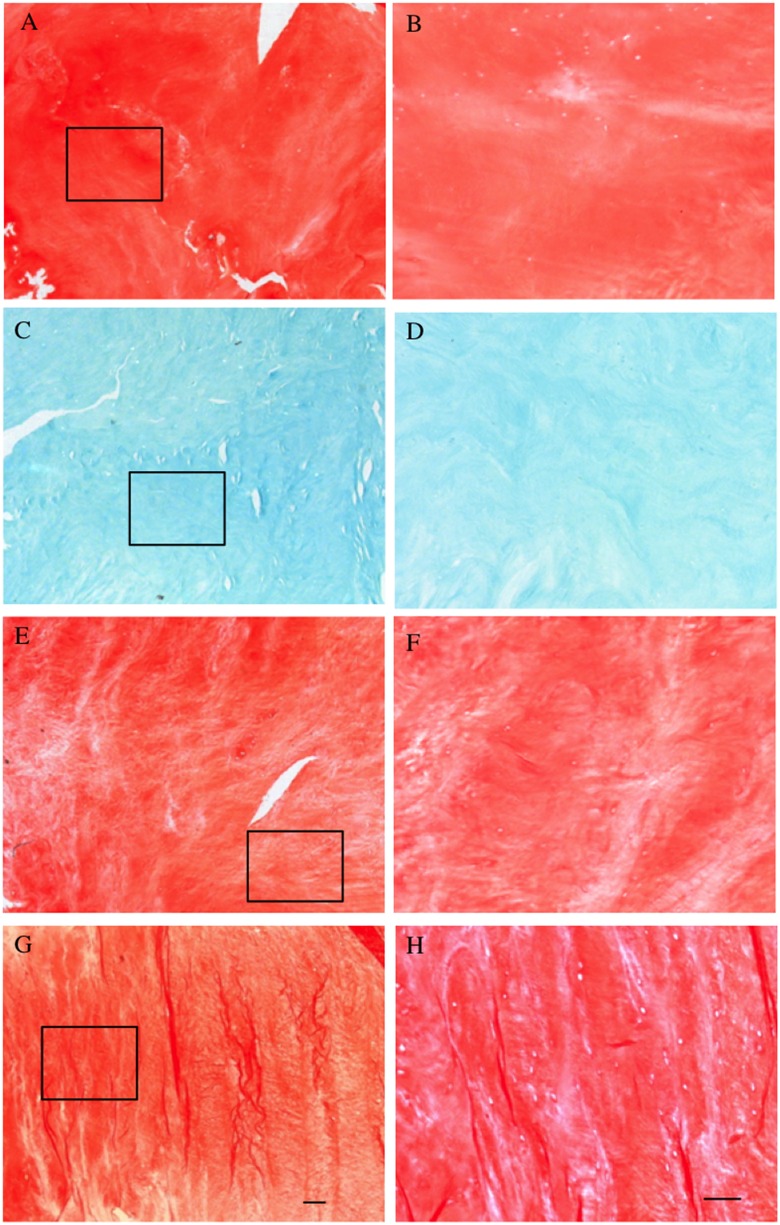
Proteoglycan distributions in loaded and unloaded discs. Discs were cultured for 14 days unloaded (A–D) or loaded (E–H), without (A, B, E and F) or with trypsin-induced degeneration (C, D, G and H). The discs were evaluated by histology using Safranin O staining. A, C, E and G are show overviews of the NP region at low magnification. B, D, F and H represent a higher magnification of the boxed areas. Scale bars, 100 and 200 µm.

To evaluate proteoglycan distribution within the tissue, Safranin O and fast green staining were performed on 5 µm tissue sections. Very little Safranin O (red) staining was observed in the unloaded trypsin-treated samples, whereas control discs and trypsin-treated loaded discs showed a strong Safranin O staining (Figure 8). The results further confirmed that the proteoglycan content in the trypsin-treated unloaded discs was depleted throughout the NP region. In the trypsin-treated loaded discs, the intensity and distribution of the Safranin O staining showed an even distribution throughout the NP region, similar to that of control discs, thus confirming the presence of metabolically active cells able to restore the tissue proteoglycan content throughout the extracellular matrix.

## Discussion

Proteoglycan depletion is the first stage of disc degeneration and may be reversible with appropriate cellular stimulus. Physiological load could be such a stimulus. Trypsin was injected into the center of bovine discs to induce proteoglycan loss [27] and model early disc degeneration. The discs were then cultured in a bioreactor [19] under a cyclic dynamic loading scheme mimicking physiological load or cultured with no load for 14 days. After this time the discs exhibited excellent cell viability and metabolic activity. The present study indicates that physiological load has the ability to stimulate matrix synthesis and fully restore proteoglycan content in bovine discs following trypsin-induced depletion. This is, to our knowledge, the first report to demonstrate that physiological load has reparative effect in IVDs with mild degeneration.

Disc degeneration with back pain is a leading cause of disability and loss of workplace productivity in otherwise healthy young adults [33]. The degenerative changes start early in life and progress with advancing age [34]. Nutrient deprivation is one factor that has been suggested to contribute to disc degeneration [35]. It may lead to cellular senescence and decreased matrix production, particularly proteoglycan synthesis, and an increase in protease production resulting in degradation of the IVD matrix [13], [14]. Studies using isolated non-degenerate discs cultured in bioreactors have confirmed a detrimental effect of low nutrient supply on cell viability [25] and shown that limited nutrition and high frequency loading had synergistic effects resulting in increased cell death and a metabolic shift with increased MMP13 gene expression [24]. Excessive mechanical load magnitude is another factor implicated in disc degeneration. It is clear from the literature that excessive axial or wedged compression, especially in combination with torsion, has a negative effect on IVD homeostasis [17], [26], [36]. Complex excessive compression resulted in decreased cell viability and increased catabolism [17], [36]. However, the effect of physiological loading has been much less studied and there are no studies describing the effect of physiological load in mildly degenerated discs.

A model of early degeneration is necessary in order to study the effect of load during this phase of degeneration. A number of methods using relatively high concentrations of proteases to induce degeneration in larger discs *ex* *vivo* have been described in the literature. Most of them were created to induce a more severe degeneration with the formation of a cavity so allowing injectable hydrogels to be evaluated [37]–[39]. We previously described a protocol to induce mild disc degeneration using trypsin [27], resulting in proteoglycan depletion but not in the formation of a cavity so more accurately mimicking the situation in early disc degeneration. In the present study we increased the trypsin dose to create a somewhat more severe degeneration. However, the doses chosen were still low enough to maintain the weigh-bearing capacity of the discs and it did not result in a depletion of viable NP cells. It was important to maintain a metabolically active cell population in the NP region allowing us to evaluate the cellular effect of physiological load on matrix synthesis in IVDs with mild degeneration.

Chondroadherin is an important indicator of matrix homeostasis, as it is fragmented in degenerate adolescent scoliotic and adult human discs possibly as a result of excessive loading [10], [11]. Chondroadherin is known to provide cell attachments in the matrix via integrin and syndecan receptors, thereby mediating mechanical changes between the cell and extracellular matrix [5], [6], [8]. Chondroadherin remained intact in both loaded and unloaded discs with a non-significant increase in abundance in the loaded discs, further supporting a that the load applied to these discs is in a physiological range.

The healthy adult human spine sustains a wide range of compressive loads during daily activity ranging from 0.1–0.3 MPa, up to 1 MPa and more than 2 MPa while lifting a 20 kg weight with a rounded back. A loading regime mimicking a relatively sedentary lifestyle was chosen for this study. The loading regime mimics a daily regime with 2 h light physical activity in the morning followed by 6 h of a low activity work environment, another 2 h of light physical activity in the afternoon, and finally a longer rest period during the evening and night. We compared this to a situation mimicking complete bed-rest where no cyclic dynamic load was applied. We have previously found that bovine tail discs are less adapted than human IVDs to withstand cyclic dynamic load in our bioreactor system [19]. A very clear negative effect on cell viability was found already at 0.6 MPa, whereas cell viability was maintained under no load or 0.3 MPa load magnitude. Thus 0.3 MPa was used as a physiologically relevant load magnitude in the present work.

The importance of low frequency dynamic loading for IVD homeostasis is clear from the results. In addition to a restoration of the proteoglycan content throughout the NP region newly synthesized extractable type II collagen was significantly increased in the loaded discs. These molecules represent the major components of the healthy IVD extracellular matrix, with the swelling potential of aggrecan and tensile properties of collagen together providing a healthy disc with its resistance to load. Taken together our data demonstrate that tissue homeostasis in mildly degenerate IVDs is strongly improved by physiological load. Such knowledge is important for patient advice on lifestyle following a biological repair procedure for mild disc degeneration. It is also important to stress the value of physiological activity in young individuals in maintaining healthy discs.
